# Survival of pediatric patients with ependymoma in a tertiary cancer center in Rio de Janeiro, Brazil

**DOI:** 10.3389/fonc.2024.1296636

**Published:** 2024-10-25

**Authors:** Gabriela Oigman, Yung Gonzaga, Marcio Christiani, Denise Magalhaes, Veronica Moreira, Diana S. Osorio, Sima Ferman

**Affiliations:** ^1^ Division of Pediatric Oncology, National Cancer Institute, Rio de Janeiro, Rio de Janeiro, Brazil; ^2^ Division of Hematology, National Cancer Institute, Rio de Janeiro, Rio de Janeiro, Brazil; ^3^ Division of Neurosurgery, National Cancer Institute, Rio de Janeiro, Rio de Janeiro, Brazil; ^4^ Division of Radiation Oncology, National Cancer Institute, Rio de Janeiro, Rio de Janeiro, Brazil; ^5^ Division of Pathology, National Cancer Institute, Rio de Janeiro, Rio de Janeiro, Brazil; ^6^ Department of Pediatrics, The University of Texas MD Anderson Cancer Center, Houston, TX, United States

**Keywords:** childhood cancer, ependymoma, survival analysis, low-and-middle-income country, epidemiology

## Abstract

**Introduction:**

Ependymoma is the third most frequent central malignant nervous system tumor in the pediatric age group. There is scarce data in the literature on survival of these patients, especially in upper and lower middle-income countries. We aimed to describe the clinical and demographic characteristics, treatment, and outcome of pediatric patients with ependymoma admitted to a public cancer hospital.

**Methods:**

Retrospective analysis of medical records of patients with ependymoma, admitted to the Pediatric Oncology department (0-20 years) during the period of 2000-2022. Data on patient, disease characteristics, and treatment were analyzed. Overall survival (OS) was calculated using the Kaplan-Meier method.

**Results:**

Seventy-two patients were evaluated; median age at diagnosis was 6.5 years (range: 1-20), 63% were male, 54% of the tumors were in the posterior fossa (PF-EPN), 45% were classified as WHO grade 3, and 68% were operated on in other institutions before referral. Regarding treatment, 72% underwent radiotherapy and 33% of patients underwent chemotherapy. Almost 70% percent of the patients had relapses. The median follow-up time was 5.2 years (Range: 0,1-21,4). The OS in 5 years was 67%. Totally resected tumors had OS in 5 years of 88% (p: 0.028).

**Conclusion:**

The results achieved in this series show a survival gap between UMIC and HIC. Relapses occurred mainly in the first ten years and then reached a plateau, with the majority of patients experiencing endocrinological and neurological sequelae.

## 1 Introduction

Ependymoma is the third most frequent pediatric malignant brain tumor and the most common tumor in spinal cord and cauda equina. Patients can often experience multiple recurrences and poor long-term overall survival (OS) (1, 2). According to CBTRUS (the Central Brain Tumor Registry of the United States), the average annual age-adjusted incidence rate is 0.29 per 100,000 for children and adolescents (0-19 years), and is higher among younger children (0-4 years old) at 0.46 per 100,000, lowering with increasing age to 0.26 per 100,000 (15-19 years) (2). High-income countries (HIC) report 5-year OS of 80-85%, while there is scarce information on survival outcomes in low-to-middle income countries (LMIC), but reports range from 40-60% (3–5). Delayed diagnosis, lack of specialized professionals, toxic death, shortage of chemotherapies, and limited infrastructure are some of the barriers that LMIC have been facing.

Historically, treatment of Central Nervous System (CNS) tumors has been neglected because of the complexity required for diagnosis and treatment. Health systems are required to have optimized referral systems from primary care or emergency departments to cancer centers where patients can be assisted by a specialized team (6).

Standard treatment has not changed much over the years whereby a maximal, safe surgical resection is still considered the most important prognostic factor in ependymoma followed by the administration of focal radiation for a large number of cases, even in young children (7–9). Chemotherapy remains a controversial option because of the questionable chemosensitivity of ependymomas. In the context of post-operative residual disease, it can be delivered pre-irradiation, although with contradictory results (10–12). The role of maintenance chemotherapy (after surgery and radiation) was investigated by COG ACNS 0831 protocol, the final publication of which is pending (13).

Our retrospective review aimed to describe the clinical and demographic characteristics, treatment, and survival outcomes of pediatric patients with ependymoma admitted to a LMIC public cancer hospital over 20 years.

## 2 Material and methods

### 2.1 Study population

A retrospective study was conducted at the National Cancer Institute (INCA), located in Rio de Janeiro, Brazil. Demographic, clinical, disease, and treatment characteristics were retrieved from medical records of pediatric patients with ependymoma, admitted to the Pediatric Oncology Department during the period of 2000-2022. We included any patient less than 20 years of age at admission with a confirmed diagnosis by histopathology of ependymoma from any location within the central nervous system in the newly diagnosed and recurrent setting. Patients previously operated on and treated with chemotherapy outside of INCA were eligible.

### 2.2 Statistics

The median and interquartile range were used to summarize the quantitative variables, and absolute and percentage values were used for the categorical variables. Survival curves were generated using the Kaplan-Meier method and statistically compared using the log-rank test. All analyses were performed using the statistical software R, version 3.6.3 (2020-02-29). Patients with myxopapillary ependymoma were excluded from the survival analysis because they are a distinct histology. Patients not seen at the institution for more than two years were considered lost to follow up and were censored. Analysis was performed on December 12, 2023.

### 2.3 Setting

According to the World Bank, Brazil is an UMIC (upper middle-income country), with a population of 215 million inhabitants and a Gross Domestic Product (GDP) per capita of US$ 8,900 (14). INCA is a tertiary cancer center and accounts for the treatment of most pediatric CNS tumors in Rio de Janeiro with free care, provided by Brazil´s Unified System of Care (SUS). The Ministry of Health is responsible for the development and coordination of integrated actions in the prevention and control of cancer (15). Today it is equipped with a pediatric inpatient ward with 22 beds, an intensive care unit (since 2002), an emergency department (since 2009), and a radiation therapy center with 3D conformal technique (since 2002). There is a large team of professionals dedicated to pediatric care: pediatric oncologists, pediatric neurosurgeons, pathologists, radiologists, radiation oncologists, physiotherapists, speech therapists, nutritionists, an abandonment prevention team, and a clinical research team, among others. Pediatric supportive care is available from the pediatric emergency and pediatric intensive care unit. Around 40 new patients with CNS tumors are treated annually at INCA.

### 2.4 Study definitions and treatment

Ependymomas were divided into supratentorial ependymomas (ST-EPN), posterior fossa ependymomas (PF-EPN), and spinal ependymomas (SP-EPN). The extent of surgical resection was evaluated by MRI and/or CT of brain or spine within 48-72 hours postoperatively, according to exam availability at the institution. The extent of resection was categorized into two major groups based on the surgeon’s report and/or MRI performed at time of patient registration at the institution: gross total resection (GTR) and subtotal resection (STR)/biopsy. Metastatic disease was defined by disease outside of the primary location as seen on brain and spinal MRI and/or cerebrospinal fluid (CSF) cytology. The histopathological diagnosis was divided according to WHO grading (1, 2, and 3) and specific histological subtypes (classic, anaplastic, myxopapillary, clear cell, tanycytic, and papillary) and immunohistochemistry using EMA (epithelial membrane antigen), S100, Olig2, and GFAP (glial fibrillary acidic protein). The pathological diagnosis did not include newer immunohistochemistry and molecular studies to classify the tumors in this cohort. Patients operated on outside INCA had their histopathology confirmed at our institution. During this long period, many treatment regimens were used. Most PF-EPN received focal RT after surgery. Radiation therapy was indicated for rade 3 ST-EPN, irrespective of their extent of resection, and all grade 2 partially resected ST-EPN. Intracranial tumors were treated with different doses: anaplastic tumors were treated with 59.4Gy in 33 fractions of 1.8Gy and other grades received 54Gy in 30 fractions of 1.8Gy. Spinal tumors were treated with doses between 45 and 50.4 Gy, in 25 to 28 fractions. Multiple chemotherapy regimens were used during the period, with different intents: to bridge infants to radiation therapy (Baby POG) and for patients with residual disease pre-irradiation (CCG 9942). In the first ten years, ICE Protocol was the chemotherapy used in the recurrent setting. Currently the COG ACNS 0121 has been used to attempt to minimize residual disease before second-look surgery, while oral etoposide is still used for palliative treatment.

At recurrence, re-operation was attempted when feasible and re-irradiation was performed in some cases, even when surgery was not possible. OS was measured as the time from registry at INCA to the date of death or last follow-up. OS was calculated using the Kaplan-Meier method.

## 3 Results

From 2000-2022, 82 patients were admitted with ependymoma. Patients were excluded from the study due to lack of data (n=9) and change of diagnosis according to the pathology review at the institution (n=1). In total, 72 patients were eligible for analysis. Patient characteristics are described in **Table 1**. Median age at diagnosis was 6,5 years (range: 1-20), with a male predominance (62%). Sixty-eight percent of patients were primarily operated on in other institutions before referral to INCA. Four patients were registered at recurrence. There were 39 patients with PF-EPN (54%) and 24 patients with ST-EPN (33%). Eight patients had SP-EPN primaries (11%), with four myxopapillary, one clear cell, one tanycytic, one anaplastic, and one without histological subtype. Grade 3 was the histology in 45% of patients. Regarding histopathology of ST-EPN, four were WHO grade 2 and 13 were WHO grade 3 and all but two were localized. PF-EPN was localized in 23 patients (58%); 16 patients had WHO grade 2 and 13 patients were WHO grade 3.

**Table 1:** Disease and treatment information.

N: 72	n (%)
**Age (median and range)**	6,5y (1-20)
Sex	
**Male**	45 (62)
**Female**	27 (38)
Surgery location	
**INCA**	23 (32)
**Other hospitals**	49 (68)
Tumor location	
**ST-EPN**	24 (33)
**PF_EPN**	39 (54)
**SP-EPN**	8 (11)
**NI**	1 (2)
Extent of disease	
**Local**	48 (66)
**Disseminated**	9 (13)
**NI**	15 (21)
WHO Grade	
**Grade I**	4 (6)
**Grade II**	24 (33)
**Grade III**	32 (45)
**NI**	12 (16)
CSF	
**Positive**	3 (4)
**Negative**	33 (46)
**Not performed**	34 (47)
**Inconclusive**	2 (3)
Extent of resection	
**GTR**	28 (38)
**STR**	23 (32)
**NI**	21 (30)
Radiotherapy	
**Sim**	52 (72)
**Não**	20 (28)
Chemotherapy	
**Yes**	23 (32)
**N**	47 (66)
**NI**	2 (1)
Recurrence/progression	
**Sim**	50 (70)
**Não**	22 (30)

CSF, Cerebrospinal fluid; GTR, Gross total resection; STR, Subtotal resection; RT, radiation therapy; CT, chemotherapy; NI, not informed.

Only two patients received a brain MRI within 48h post-operative, with the remaining patients being submitted to post-operative CT scan. Only 54% of patients had craniospinal MRI (pre- or post-operatively). Of these, 26% had spine MRI within one month of surgery. Cerebrospinal fluid (CSF) was assessed for neoplastic cells in 38 patients (52% of cases) with positivity in three patients (8%). Resection grade reports (either by MRI reports or surgeons report) were available in 70% of patients, namely 38 patients with gross total macroscopic resection. After first surgery, 52 patients received focal radiation. One other patient received craniospinal radiation at recurrence. Eleven patients were not submitted to RT at any moment. Of these, four patients remained alive.

Only 22 patients (30%) received CT: 15 PF-EPN (68%), six ST-EPN (27%), and one SP-EPN (5%). Eleven patients received CT for adjuvant treatment (including three infants treated with bridge therapy, as per Baby POG and BB SFOP), and four patients before second-look surgery, as per COG ACNS 0121), six patients received ICE protocol, and five patients received oral chemotherapy with palliative intent (oral etoposide). Radiation therapy after surgery was administered in 52 patients: 19 ST-EPN (36%), 27 PF-EPN (52%), and six SP-EPN (12%). Five ST-EPN patients were initially just observed.

Fifty patients (70%) had relapses: 17 patients with ST-EPN (34%) and 27 patients with PF-EPN (54%). Salvage treatments included at least one additional re-resection in 20% of patients and re-irradiation in 15% (all focal and one craniospinal).

At the time of analysis, 28 patients were alive and 22 of these had some degree of long-term sequelae (78%). Only six patients (22%) did not present any sequelae. The most common were neurological, in 67% of patients, with the following symptoms: cerebellar ataxia, intellectual deficit, epilepsy, facial palsy, dysphagia, hypotonia, and strabismus. Twenty-five percent of patients also had endocrinological symptoms, with growth hormone and thyroid deficiencies being the most common.

**Figure 1:**
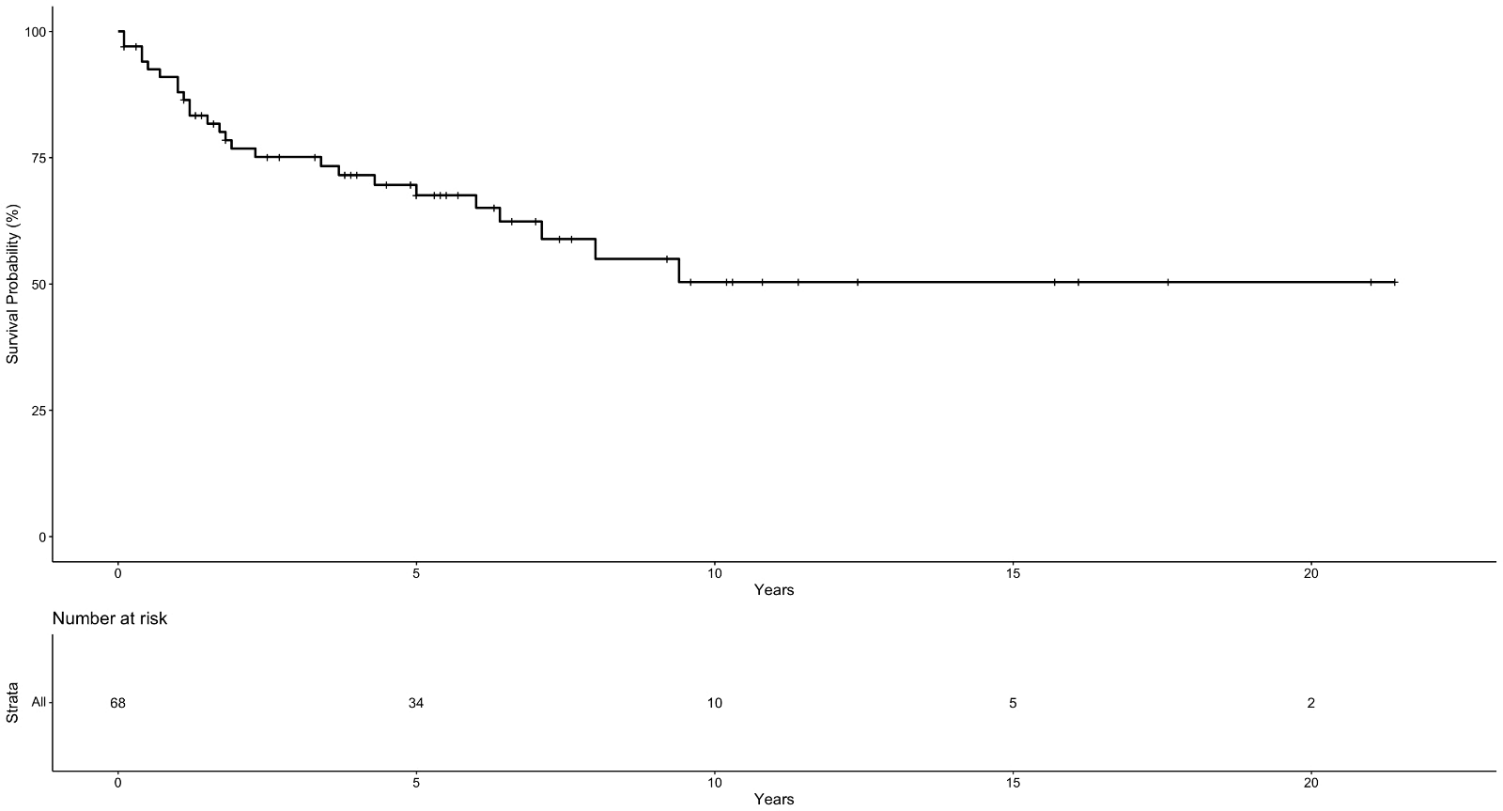
Ependymoma Overall Survival Probability.

**Figure 2:**
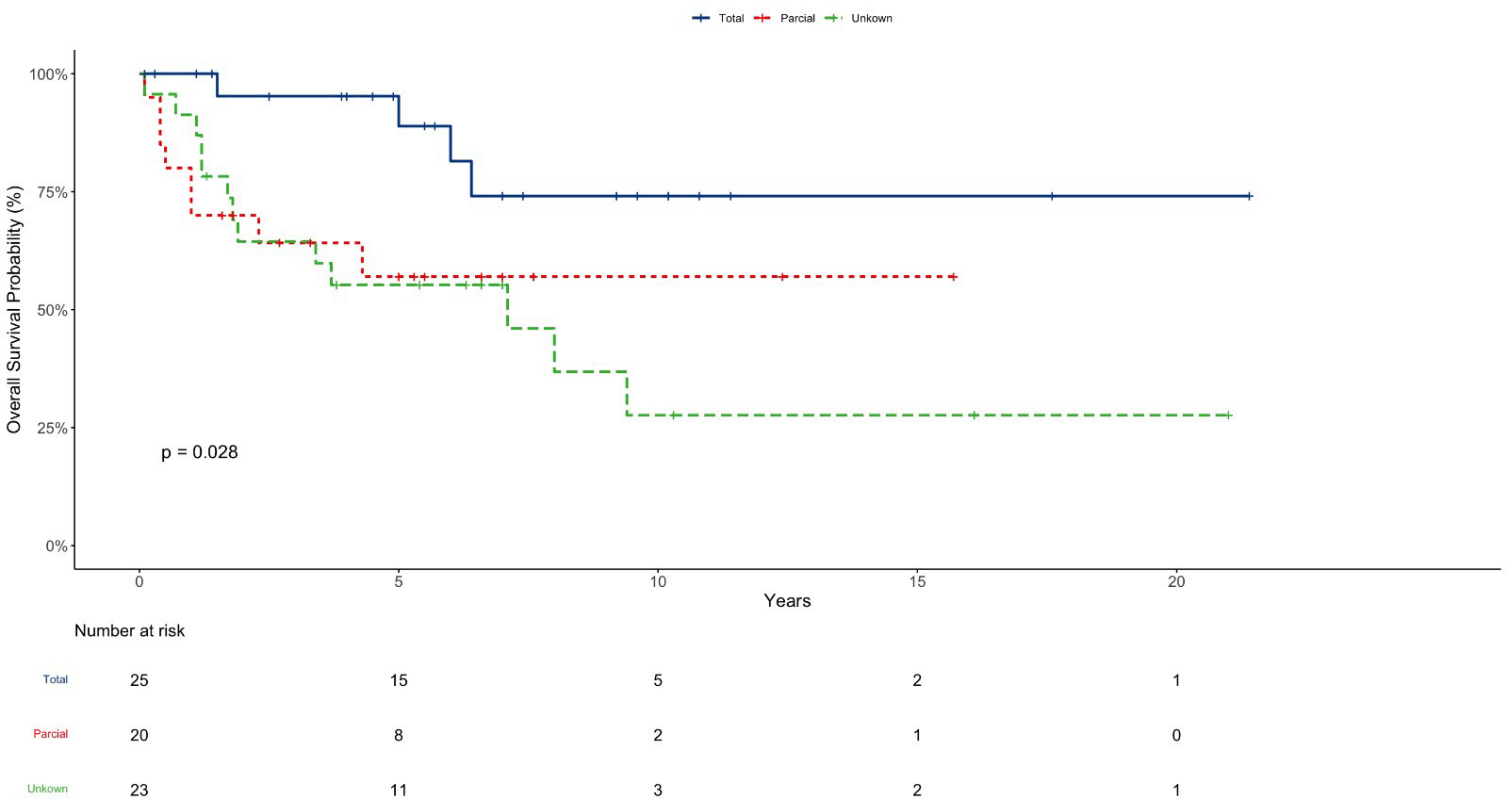
EP OS according to resection grade.

**Figure 3:**
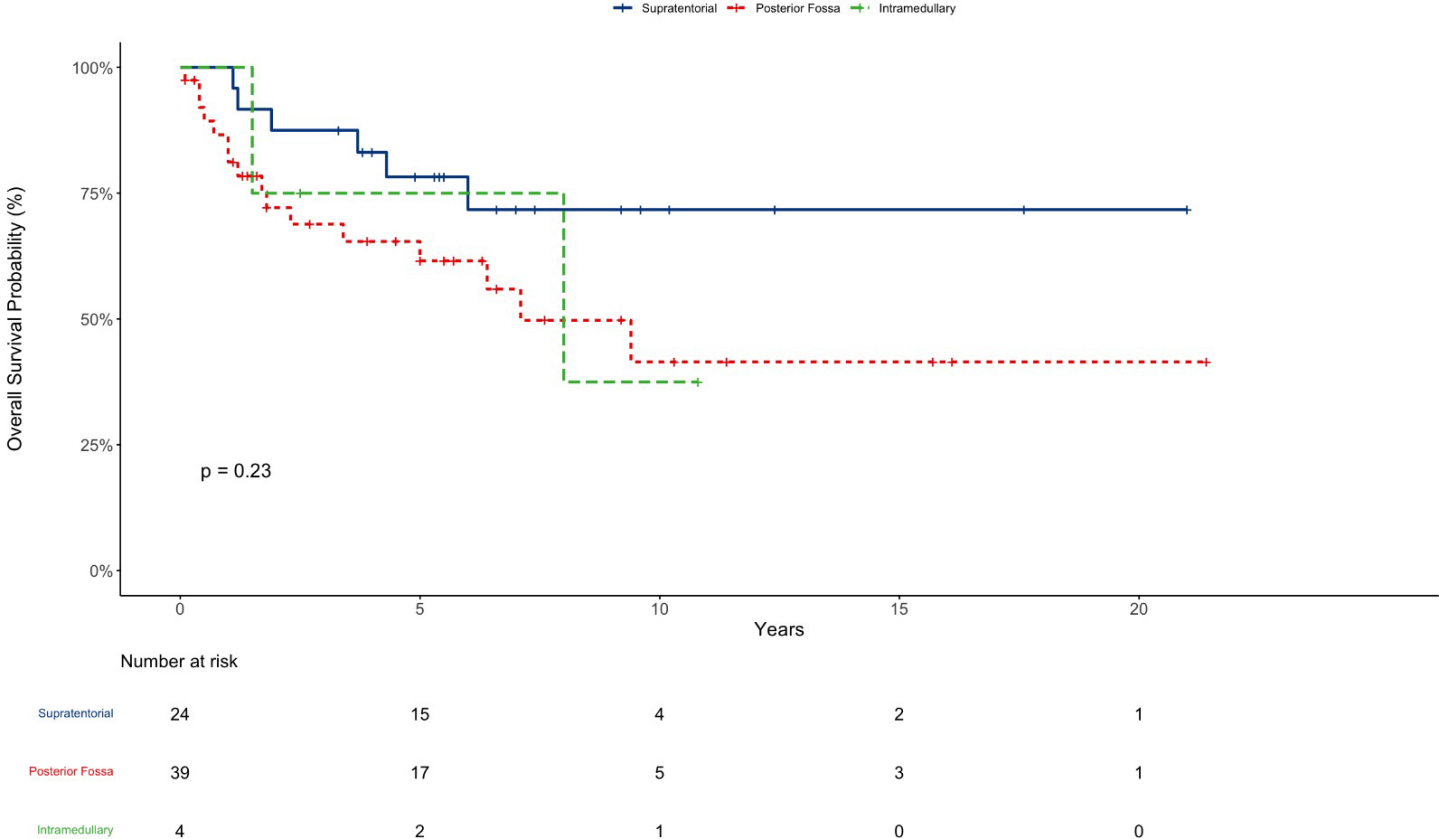
EP OS according to tumor location.

**Figure 4:**
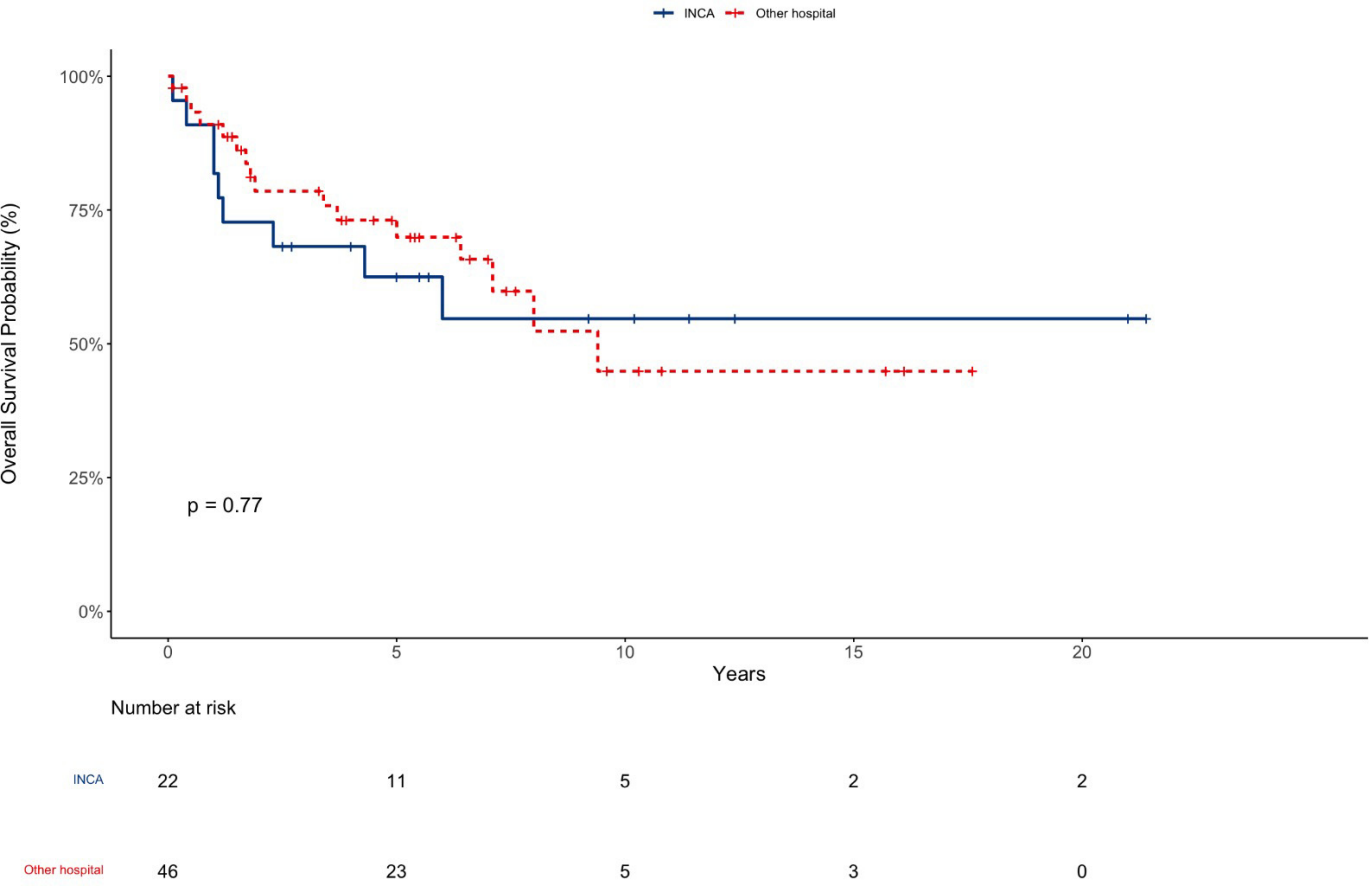
EP OS according to place of surgery.

The median follow-up time for this cohort was 5.9 years (Range: 0,1-21,4); 14 patients were lost to follow up. The OS in 5, 10, and 20 years was 67%, 50%, and 50% respectively with 34, five, and two patients surviving 5, 10, and 20 years respectively (**Figure 1**). The OS in 5 years for patients with totally resected tumors was 88% and for partially resected was 57% (p: 0.028) (**Figure 2**). Regarding tumor location, OS in 5 years for ST-EPN was 78%, PF-EPN 61%, and SP-EPN was 75% (**Figure 3**). The OS in 5,10, and 20 years for patients submitted to surgery at INCA was 62%, 54%, and 54%, and for patients with surgery elsewhere was 69%, 44%, and 51%, respectively (p: 0.77) (**Figure 4**).

## 4 Discussion

Although the overall survival rate at 5 years of 67% was satisfactory, the long-term results were poor and the rate of recurrences higher than in HIC. This highlights that current treatment in LMIC settings should be improved. **Table 2** shows the comparison of OS and EFS in countries of different economic status according to the World Bank. There are a few Brazilian papers, and the newest one, focusing on posterior fossa tumors, showed an OS in 5 years of 49% (23); other Brazilian studies in intracranial ependymomas reported OS of 60% and 33% (24, 25). HIC show OS around 82% (10, 26), whereas UMIC have similar survival rates to the present cohort (4, 27).

**Table 2:** Comparative survival.

Citation	Country	Economic status	Number of patients	Population	Tumor location	OS (%)	EFS (%)
**De Andrade (2009) (** 16)	Brazil	Upper middle income	34	Ped and adults	Intracranial/ spinal	60 (5y)	
**De Araujo (2011) (** 17)	Brazil	Upper middle income	8	pediatric	Intracranial	33 (5y)	
**Godfraind (2012) (** 18)	USA	High income	146	pediatric	Intracranial	82	69
**Tashvighi (2018) (** 5)	Iran	Lower middle income	73	pediatric	Intracranial	61 (3y)	59 (3y)
**Wang (2018) (** 19)	China	Upper middle income	55	Ped and adults	Intracranial	64 (5y)	49 (5y)
**Das (2018) (** 3)	India	Lower middle income	20	pediatric	Intracranial		35 (3y)
**Ruangkanchanasetr (2019) (** 4)	Thailand	Upper middle income	24	pediatric	Intracranial	75	56
**Shah (2020) (** 20)	Saudi Arabia	high income	22	pediatric	Intracranial	44	18
**Hammad (2021) (** 21)	Egypt	Low income	47	pediatric	Intracranial	43 (3y)	43 (3y)
**Ritzmann (2022) (** 8)	UK, Ireland, Spain, Denmark, Sweden, Netherlands	high income	72	pediatric	Intracranial	69 (5y)	49,5 (5y)
**Da Costa (2023) (** 22)	Brazil	Upper middle income	55	pediatric	Intracranial (Posterior fossa)	49 (5y)	

OS, Overall survival; EFS, Event free survival.

Treating pediatric CNS tumors in low- and middle-income countries can be very challenging, as they require complex care with multidisciplinary teams comprising pediatric oncologists, pediatric neurosurgeons, neuropathologists, neuroradiologists, radiation therapists, and technology and clinical support, which is not always accessible (28). With epidemiological transition, chronic diseases such as cancer have become leading causes of death. CNS tumors are the first cause of disease-related mortality in pediatric solid tumors in Brazil, with specific adjusted mortality rate of 10,26 per million of children/adolescents, according to INCA (29). Barriers to care, such as delayed diagnosis, treatment abandonment, malnourishment, and low parental education, explain the survival gap faced by children in this setting (30, 31). Currently, pediatric neuro-oncology experts have been addressing how to bridge the gap with interventions, for instance, twinning programs, optimization of available resources, and establishment of multidisciplinary teams (20, 32).

Ependymomas are surgical tumors, with questionable response to current chemotherapy protocols (33). Also, the prognosis is related to the extent of resection (34), with patients with totally resected tumors having better survival than patients with residual tumors (35). Ependymomas are sharply demarcated tumors, usually rising from the fourth ventricle, but specific locations (cerebellopontine angles and eloquent areas, for example) may eventually be a deterrent to gross total resections. The shortage of subspecialized pediatric neurosurgeons can directly impact the grade of resection in brain tumors and, therefore, the survival (36). According to Brazilian pediatric neurosurgery society (SBNPed), there are 143 Brazilian pediatric neurosurgeons (37) for a population of around 62 million children/adolescents under 19 years (22). Of those, 84 pediatric neurosurgeons are concentrated in the southeast region (comprising Rio de Janeiro, Sao Paulo, Minas Gerais, and Espirito Santos). In Rio de Janeiro, where INCA is located, there are 19 pediatric neurosurgeons for a population of 4,6 million children/adolescents under 19 years (16), with a pediatric neurosurgeon for every 245,000 children, which is higher than other LMICs with one pediatric neurosurgeon for every 3.6 million children (17). In the present study, 68% of patients had first tumor surgery outside INCA, including emergency hospitals, and were than referred for adjuvant treatment. It is not possible to identify if they were operated on by pediatric neurosurgeons, however it is described that pediatric neurosurgeons are more prone to remove above 90% of the tumors (36). Therefore, we encourage the transfer of brain tumor patients to INCA and all our efforts are towards reoperation in case of residual tumor on imaging.

Although long-term OS for patients operated on at INCA was superior, it was not statistically significant. Due to the small cohort numbers, it was not possible to accurately assess this difference.

In this series, only 51 patients had reports on extent of resection, and of these, 28 patients had gross total resection (54%) with the following locations: 11 supratentorial tumors, 12 posterior fossa tumors, and five spinal tumors. Extent of resection was significant to survival. Other studies show similar results (19, 38).

Adequate pre- and post-operative imaging with brain and spinal Magnetic Resonance Imaging (MRI) is the gold standard imaging evaluation for these tumors. Postoperative imaging guidelines for brain tumors recommend that brain and spine MRI should be performed within 48-72h hours to define the extent of resection (18). Unfortunately, not every hospital in our setting has an MRI and some children in our study were registered without appropriate post-operative imaging tests. In this series, only two patients had brain MRI within 48h post-operative, and the remaining patients were submitted to post-operative CT scans. Twenty-six percent had spine MRI within one month of surgery. Instituting imaging protocols (early post-operative brain MRI) in intensive care units is mandatory to properly define grade of resection and to program further surgery in order to have no residual tumor.

Interventions to achieve better surgical results, besides the pediatric neurosurgical specialization, are technological improvements like intraoperative neurophysiological monitoring. This technique assesses the integrity of cranial nerves, allowing safer surgeries and larger resections with less neurological morbidities, but are rarely available in LMIC settings because of the high cost (21).

This series reports more than 20 years of treatment with different treatment strategies. Patients received several chemotherapy protocols (10, 11, 39–41): to delay radiation therapy, before second-look surgery, and for palliation. Currently, patients above one year have been receiving focal radiation therapy after surgery, instead of chemotherapy, since it improves survival (42). For recurrent disease there is no standard salvage treatment. Regarding re-irradiation, all patients received focal radiation therapy, except one who received CSI. Currently, CSI re-irradiation has shown improvement in survival in recurrence (43, 44).

Ependymomas are tumors with high recurrence rates, and even with standard therapies one third of patients fail treatment (43). In this series, 70% of patients recurred once, with multiple salvage treatments in different combinations: surgery, irradiation (or reirradiation), and chemotherapy,. Although there were more relapses than described in the literature, the overall survival of this series was similar to other upper middle-income countries.

Long-term sequelae were found in 80% of ependymoma survivors in this study, with neurological and endocrinological alterations being the most common. Hormone replacement was indicated when necessary. Patients with neurological deficits were followed by pediatric neurologists, speech therapists, physiotherapists, and occupational therapists. These numbers highlight the need to improve not only treatment, with the aim of increasing survival, but also paying attention to patient quality of life. Vulnerable patients such as ependymomas survivors in countries with limited resources must be submitted to neuropsychological assessments. Initiatives such as The European Society of Paediatric Oncology Ependymoma-II program Core-Plus model for an internationally accepted test battery for follow-up of pediatric ependymoma patients has been developed (45, 46).

Study limitations include the retrospective nature of the study, long period of inclusion of patients with different treatment strategies, lack of neuropsychological data, and lack of imaging results in some patients, mostly in the early years. The strength of the study is a cohort of patients from the same institution.

In conclusion, with a multidisciplinary approach, survival outcomes were similar to those described in literature for upper-middle-income countries, but still less than those achieved in HIC. Relapses occurred mainly in the first ten years and then reached a plateau, with the majority of patients experiencing endocrinological and neurological sequelae. There is still a need for improvement, with earlier referral to specialized hospitals, more imaging studies to define grade of resection, more reoperation, and timely adjuvant treatment, when indicated.

## Data availability statement

The original contributions presented in the study are included in the article/supplementary material. Further inquiries can be directed to the corresponding author.

## Ethics statement

Ethical review and approval was not required for the study of human participants in accordance with the local legislation and institutional requirements. Written informed consent from the patients/participants or patients/participants’ legal guardian/next of kin was not required to participate in this study in accordance with the national legislation and the institutional requirements.

## Author contributions

GO: Writing – original draft. YG: Data curation, Writing – review & editing. MC: Conceptualization, Writing – review & editing. DM: Conceptualization, Writing – review & editing. VM: Conceptualization, Writing – review & editing. DO: Supervision, Conceptualization, Formal analysis, Writing – original draft, Writing – review & editing. SF: Writing – original draft.
